# Using Central Composite Experimental Design to Optimize the Degradation of Tylosin from Aqueous Solution by Photo-Fenton Reaction

**DOI:** 10.3390/ma9060428

**Published:** 2016-05-30

**Authors:** Abd Elaziz Sarrai, Salah Hanini, Nachida Kasbadji Merzouk, Djilali Tassalit, Tibor Szabó, Klára Hernádi, László Nagy

**Affiliations:** 1 Laboratory for Biomaterials and Transport Phenomena LBMPT, University Yahia Fares, Medea 26000, Algeria; s_hanini2002@yahoo.fr; 2 Unité de Développement des Equipements Solaires, UDES/Centre de Développement des Energies Renouvelables, CDER, Bou Ismail, Tipaza 42415, Algeria; nkmerzouk@gmail.com (N.K.M.); tassalit2003@gmail.com (D.T.); 3 Department of Medical Physics and Informatics, University of Szeged, Szeged 6720, Hungary; tiberatosz@gmail.com (T.S.); L.Nagy@physx.u-szeged.hu (L.N.); 4 Department of Applied and Environmental Chemistry, University of Szeged, Szeged 6720, Hungary; k.hernadi@chem.u-szeged.hu

**Keywords:** Photo-Fenton, Tylosin, RSM, CCD

## Abstract

The feasibility of the application of the Photo-Fenton process in the treatment of aqueous solution contaminated by Tylosin antibiotic was evaluated. The Response Surface Methodology (RSM) based on Central Composite Design (CCD) was used to evaluate and optimize the effect of hydrogen peroxide, ferrous ion concentration and initial pH as independent variables on the total organic carbon (TOC) removal as the response function. The interaction effects and optimal parameters were obtained by using MODDE software. The significance of the independent variables and their interactions was tested by means of analysis of variance (ANOVA) with a 95% confidence level. Results show that the concentration of the ferrous ion and pH were the main parameters affecting TOC removal, while peroxide concentration had a slight effect on the reaction. The optimum operating conditions to achieve maximum TOC removal were determined. The model prediction for maximum TOC removal was compared to the experimental result at optimal operating conditions. A good agreement between the model prediction and experimental results confirms the soundness of the developed model.

## 1. Introduction

During the last two decades, an increasing interest has been shown in the ecological effects of pharmaceuticals and personal care products, which are released into the environment every year [1,2]. Antibiotics are a special group of pharmaceutical compounds used to control infectious diseases in human and veterinary medicine. A residual concentration has been detected in various environmental compartments worldwide due to the fact that a large portion of the consumed antibiotics are not completely metabolized (and thus are excreted as active substances) and the conventional wastewater treatment methods fail to completely remove them from the solution [3,4,5]. Their presence in aquatic systems increases the resistance of bacteria to the antibiotic functions of these chemicals, raising great concern about their transport, fate, ecological effects and risk in the environment [6,7,8]. The existence of antibiotics in natural water bodies poses serious threats to human health. Besides human health, toxic effects of antibiotics on aquatic and edaphic organisms also pose an ecological risk [9,10,11]. The environmental exposure of antibiotics could increase the possibility of changes in the microbial populations to degrade contaminants such as pesticides [12], and have a deleterious effect on important biogeochemical cycles such as nitrification and denitrification [13].

**Figure 1:**
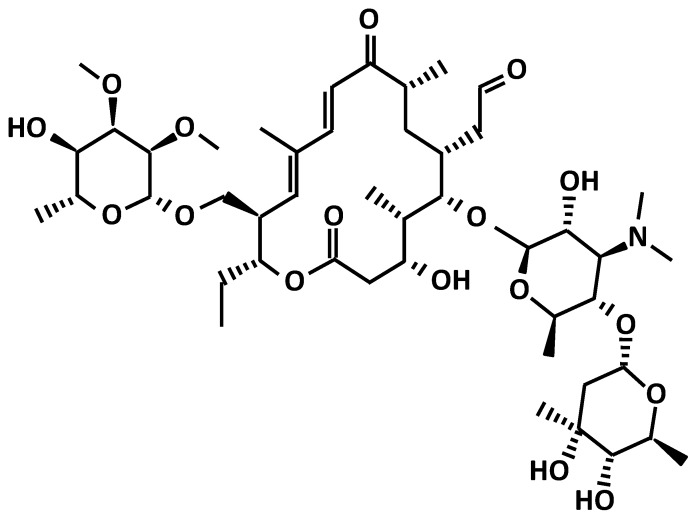
Chemical structure of Tylosin.

Among veterinary pharmaceuticals, Tylosin (Figure 1) is a macrolide antibiotic produced by the fermentation of Streptomyces strains. It consists of a substituted 16-membered lactone, an amino sugar (mycaminose) and two neutral sugars, mycinose and mycarose. Tylosin is used extensively as a therapeutic substance in the treatment of mycoplasmosis in poultry and livestock [14].

Fenton and photo-Fenton are practical advanced oxidation processes, used for treating wastewater containing pharmaceutical products, in particular antibiotics [15]. The Fenton process (Fe^2+^/H_2_O_2_/dark) (Equations (1)–(6)) is a homogeneous catalytic oxidation process that uses a mixture of H_2_O_2_ and Fe^2+^ in an acidic environment; the reaction between dissolved Fe^2+^ and H_2_O_2_ leads to the oxidation of Fe^2+^ to Fe^3+^ and the production of hydroxyl radicals (HO**^.^**) [16].
Fe^2+^ + H_2_O_2_  ⟶  Fe^3+^ + OH**^.^** + OH^−^ (1)Fe^2+^ + OH**^.^**  ⟶  Fe^3+^ + OH^−^ (2)OH**^.^** + Organics  ⟶  Products (3)OH**^.^** + H_2_O_2_  ⟶  H_2_O + H_2_O^.^ (4)OH**^.^** + OH**^.^**  ⟶  H_2_O_2_ (5)Fe^3+^ + H_2_O_2_  ⟶  FeOOH^2+^ + H^+^ (6)


These reactions show that hydrogen peroxide may be consumed when it reacts with Fe^2+^, as shown in Equation (1), producing hydroxyl radicals that will degrade organic compounds through Equation (3). Hydrogen peroxide can also react with Fe^3+^ via Equation (6), but the major drawback of the Fenton reaction is the production of Fe(OH)_3_ sludge that requires further separation and disposal [17]. The rate of reaction in the Fenton process can be further enhanced by the application of ultraviolet irradiation sources, also known as the photo-assisted Fenton system [18,19]. The photo-Fenton or photo-assisted Fenton (Fe^2+^/H_2_O_2_/light) process involves irradiation with sunlight or an artificial light, and it has shown efficiency in minimizing sludge formation and improving the degradation efficiency [17]. Applying UV irradiation to the Fenton reaction can enhance the oxidation rate of organic compounds by the photo-reduction of produced ferric ions (Fe^3+^) and ferric complexes. Ferrous ions are recycled continuously by irradiation so they are not depleted during the course of the oxidation reaction, as shown in Equation (7). The photo-reduction of ferric to ferrous ions is promoted concomitantly with the generation of additional HO**^.^**, according to Equation (8) [20].
Fe^3+^ + H_2_O  ⟶  Fe(OH)^2+^ + H^+^ (7)Fe(OH)^2+^ + һʋ  ⟶  Fe^2+^ + OH**^.^** (8)


Different parameters such as hydrogen peroxide, ferrous ion concentration and pH could affect the degradation in the photo-Fenton process. In most published studies, the effect of each variable was studied independently, with the other variables kept constant. This approach fails to consider the effects of all the parameters involved, and also the optimization of the factors that will be needed for large numbers of experiments, more time and more materials. To overcome the limitations and the disadvantages of the conventional methods, optimizing the affecting factors with Response Surface Methodology (RSM) becomes necessary. RSM as a reliable statistical tool in multivariate systems fits the studied experimental domain in the theoretical design through a response function. In this study, the Total Organic Carbon removal rate (TOC %) of Tylosin by the photo-Fenton process was investigated. The effect of hydrogen peroxide, ferrous ion concentration, pH and their interactions was evaluated using a Central Composite Design (CCD) combined with RSM. The optimal operating conditions to achieve maximum TOC % removal were obtained and validated experimentally. We are convinced that the method of analysis we present here can be useful for the investigations of new types of advanced catalytic materials. 

## 2. Material and Methods

### 2.1. Materials

Tylosin powder was obtained from Eli Lilly Export S.A. Switerzland. FeSO_4_∙7H_2_O, H_2_O_2_ (30% wt), were purchased from Sigma Aldrich chemical and were used as received. NaOH (99%) and H_2_SO_4_ (99%) used to adjust pH were supplied by EMD Chemicals and used as received. All other reagents were of analytical grade.

### 2.2. Photo-Fenton Reaction

**Figure 2:**
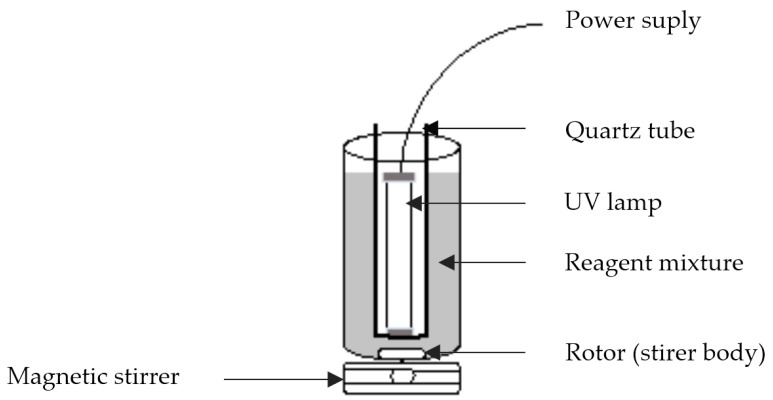
Schematic diagram of photochemical reaction device.

The 1 L bottle borosilicate glass photochemical reactor was magnetically stirred and was illuminated by a UV light lamp (type SYLVANIA, λ_max_ = 350 nm, P = 11 W, made in UK). The lamp light located vertically in the center of the reactor was used as artificial light source (Figure 2). Before the reaction started, Tylosin solution (15 mg·L^−1^) was mixed to 1 L of Milli-Q water and was homogenized for 25 min in the dark, a control sample was collected for analysis without any pre-treatment. Incorporation of FeSO_4_∙7H_2_O to the antibiotic solution was performed under permanent magnetic stirring until complete dissolution. After that, the hydrogen peroxide solution was added; finally, H_2_SO_4_ (1 mol∙L^−1^) was used to adjust the pH. In the end of the treatment, sodium sulfite anhydrous (Na_2_SO_3_) was added to stop the Fenton reaction. The dosage of Fe(II), H_2_O_2_ (30% wt) and H_2_SO_4_ was determined by the factorial design for variable optimization. Tylosin mineralization was followed by TOC analyzer Analytic Jena multi N/C 3100 TOC/TNb with the detection limit of 4 µg∙L^−1^.

All the experiments were carried out at fixed radiation time of 210 min. The experiments were performed in triplicate.

### 2.3. Central Composite Design

Response Surface Methodology (RSM) is a combination of statistical and mathematical methods used to select the best experimental conditions requiring the lowest number of experiments in order to get appropriate results [21]. A Central Composite Design (CCD) with three independent variables was applied to investigate the effect of hydrogen peroxide, ferrous ion concentration and pH on the Total Organic Carbone (TOC %) removal rate under the photo-Fenton process.

A total of 20 experiments were found to be sufficient to calculate the coefficients of the second-order polynomial regression model for three variables. Each variable was investigated at five levels: −α, −1, 0, +1 and +α, as shown in Table 1. The behavior of the UV-Fenton process is explained by the following empirical second order polynomial model (Equation (9)).

(9) $$Y\%=A_{0}+A_{1}X_{1}+A_{2}X_{2}+A_{3}X_{3}+A_{12}X_{1}X_{2}+A_{13}X_{1}X_{3}+A_{23}X_{2}X_{3}+A_{11}X_{1}^{2}+A_{22}X_{2}^{2}+A_{33}X_{3}^{2}$$

Here *Y* is the TOC reduction in % and it is calculated as follows:

(10) $$Y\%=\frac{TOC_{0}-TOC_{F}}{TOC_{0}}$$

Here *A*_0_ is the interception coefficient, *A*_11_, *A*_22_ and *A*_33_ are the quadratic terms, *A*_12_, *A*_13_ and *A*_23_ are the interaction coefficients, and *X*_1_, *X*_2_ and *X*_3_ are the independent variables studied (H_2_O_2_, pH and Fe^2+^, respectively). *TOC*_0_ and *TOC*_F_ are the Total Organic Carbon in the beginning and in the end of the reaction, respectively.

All analytical tests were carried out in triplicate. Statistical analysis was performed using the MODDE software. Data were analyzed by the analysis of variance (ANOVA), and *p*-value lower then 0.05 was considered significant in surface response analysis. The optimal values of the operation parameters were estimated by the three-dimensional response surface analysis of the independent variables (H_2_O_2_, pH and Fe^2+^) and the dependent variable (*Y*%). Range and levels of independent variables are listed in Table 1.

## 3. Result and Discussion

### 3.1. Optimal Conditions

The performance of the photo-Fenton system depends on different variables such as pH, initial iron concentration and hydrogen peroxide dosage [22]. Obviously, defining the optimal levels of all three variables would require a large number of experiments. To simplify the experimental analysis, we should understand the roles of these variables.

First, it is well known that the Fenton reaction depends strongly on the ferrous ion concentration. Some studies have reported that an increase in the concentration of Fe(III) ions increases the rate of degradation continuously and there is no optimum value [23]. However, this has not been confirmed by other studies yet [24]. There is a possible existence of a limiting catalytic concentration of Fe^2+^ above which the rate of the reaction does not increase, or it becomes lower. However, the hypothesis of application of an optimized amount of metal is very important since one of the main disadvantages associated with the homogeneous photo-Fenton reaction is the formation of large amounts of metal containing sludge at the end of the process. Such sludge does not only deliver a high environmental impact, implying additional associated costs, but these also represent the loss of significant quantities of catalytic metals [25].

Operating pH is one of the crucial factors affecting the rates of degradation, and for the photo-Fenton oxidations strongly acidic conditions are favored. A maximum catalytic activity was observed around PH = 2.8 [26,27]. This activity diminishes drastically with an increase or decrease of pH. For higher pH values, low activity is detected because of the decrease of free iron species due to ferric oxyhydroxide precipitation, formation of different complex species and breakdown of H_2_O_2_ to O_2_ and H_2_O [28,29]. Low activity at pH values, more acidic than the optimal level, results from Fe(III) forming different complex species in solution [30].

The amount of hydrogen peroxide is another parameter that influences the photo-Fenton process. It has been shown that increasing the concentration of H_2_O_2_ at optimum pH increases the rate of degradation continuously. However, there have been no reports where an optimum has been observed with respect to the hydrogen peroxide concentration beyond which the rate of degradation significantly drops [26]. 

### 3.2. RSM Model Development

In this study, the effect of three factors on the photo-Fenton process including hydrogen peroxide, pH and ferrous ion concentration were selected as factors in the Central Composite Design. As a response, the Total Organic Carbon (TOC) removal rate was chosen, a total number of 20 experiments were employed for the response surface modeling (Table 2), and the order of experiments was arranged randomly. The observed and predicted results for the percent TOC removal are also depicted in Table 2.

The MODDE software was used to calculate the coefficients of the second-order fitting equation and the model suitability was tested using the ANOVA test. Therefore, the second-order polynomial equation should be expressed by Equation (11) (conf. Equation (9)):

(11) $$Y=88.2-2.2X_{1}-11.9X_{2}+10.2X_{3}-4.2X_{1}X_{2}+4.4X_{1}X_{3}+0.5X_{2}X_{3}-0.3X_{1}^{2}-16.4X_{2}^{2}-3.2X_{3}^{2}$$

According to the monomial coefficient value of regression model Equation (11), *X*_1_ = −2.2 (H_2_O_2_), *X*_2_ = −11.9 (pH) and *X*_3_ = 10.2 (ferrous ion concentration), and the order of priority among the main effect of impact factors is pH value (*X*_2_) > ferrous ion concentration (*X*_3_) > H_2_O_2_ concentration (*X*_1_).

### 3.3. Statistical Analysis

In Table 3, the results of the analysis of variance (ANOVA) are summarized to test the soundness of the model. Analysis of variance (ANOVA) is a statistical technique that subdivides the total variation in a set of data into component parts associated with specific sources of variation for the purpose of testing hypotheses on the parameters of the model [28,31]. The mean squares values were calculated by dividing the sum of the squares of each variation source by their degrees of freedom, and a 95% confidence level (α = 0.05) was used to determine the statistical significance in all analyses.

Results were assessed with various descriptive statistics such as the *p*-value, *F*-value, and the degree of freedom (df); the determination coefficient (*R*^2^) of each coefficient in Equation (10) was determined by Fisher’s *F*-test and values of probability >*F*. As shown in Table 3, a small probability value (*p* < 0.001) indicates that the model was highly significant and could be used to predict the response function accurately. Goodness-of-fit for the model was also evaluated by coefficients of determination *R*^2^ (correlation coefficient) and adjusted coefficients of determination *R*^2^_adj_. The large value of the correlation coefficient *R*^2^ = 0.986 indicated a high reliability of the model in predicting of TOC removal percentages, by which 98.6% of the response variability can be explained by the model.

### 3.4. Effects of Model Parameters and Their Interactions

The significance of each model parameter was determined by means of Fischer’s *F*-value and *p*-value. The *F*-value is the test for comparing the curvature variance with residual variance and probability >*F* (*p*-value) is the probability of seeing the observed *F*-value if the null hypothesis is true. Small probability values call for rejection of the null hypothesis and the curvature is not significant. Therefore, the larger the value of *F* and the smaller the value of *p*, the more significant the corresponding coefficient is [32]. As shown in Table 3, we concluded that the independent variables of the quadratic model, including the pH value (*X*_2_), the ferrous ion concentration (*X*_3_) and the second-order effect of the pH value (X_2_^2^), are highly significant parameters because *p* < 0.001. Moreover, the H_2_O_2_ value (*X*_1_), the interactions between the H_2_O_2_ concentration (*X*_1_) and pH value (*X*_2_), the interactions between the H_2_O_2_ concentration (*X*_1_) and ferrous ion concentration (*X*_3_) and the second-order effect of the ferrous ion value (*X*_3_^2^) are significant because *p* < 0.05. The *p* value >0.05 means that the model terms are insignificant. We can see from Table 3 that interactions between the pH (*X*_2_) and ferrous ion concentration (*X*_3_), and the second-order H_2_O_2_ value (*X*_1_^2^) are insignificant.

**Figure 3:**
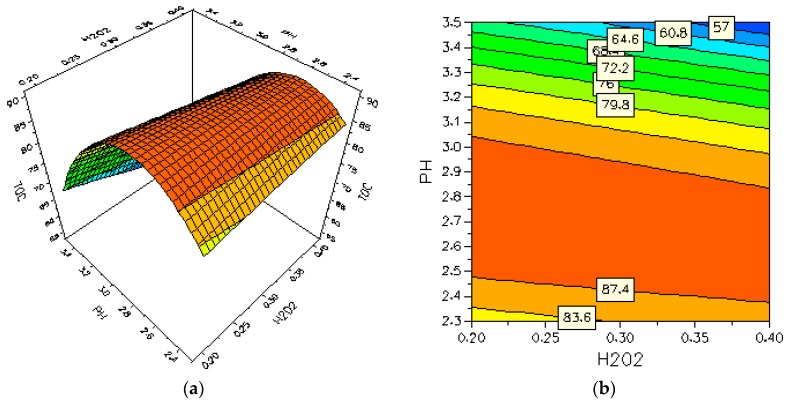

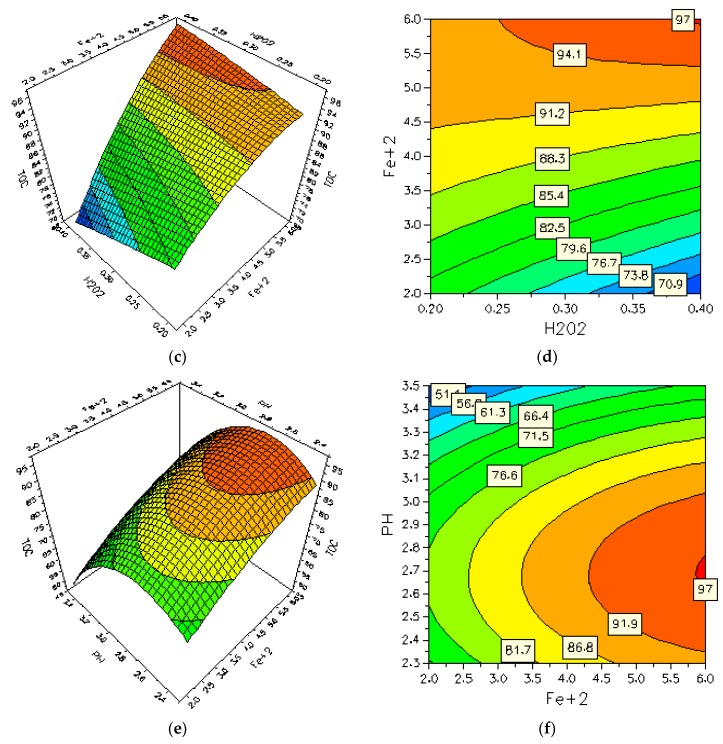
Effects of pH, H_2_O_2_ and Fe^2+^ initial concentration on TOC removal rate. (**a**,**b**) Fe^2+^ concentration was kept constant at 4 mg∙L^−1^; (**c**,**d**) H_2_O_2_ concentration was kept constant at 0.3 g∙L^−1^; (**e**,**f**) pH value was kept constant at 2.9.

The MODDE software was used to produce three-dimensional (3D) response surfaces and two-dimensional (2D) contour plots. The 3D surfaces and 2D contour plots are graphical representations of the regression equation for the optimization of reaction conditions and are the most useful approach in revealing the conditions of the reaction system. In such plots, the response functions of two factors are presented while all other factors are at the fixed levels. The results of the interactions between three independent variables and the dependent variable are shown in Figure 3.

As it can be seen in Figure 3, depending on the reaction, the H_2_O_2_ concentration, pH and Fe(II) concentration may have a positive or negative effect on the TOC removal.

Figure 3a,b shows the interaction effect of pH and H_2_O_2_ concentration on the TOC removal rate. As it can be seen in the plots, there is an increase in the TOC removal rate with an increase of pH, with the maximum TOC removal rate in the pH range of 2.48 to 3.05. Beyond this value range, the TOC removal starts to decrease with the increase of the pH. Previous studies have also reported a maximum catalytic activity around the pH range of 2.8 [28,31]. On the other hand, the effect of H_2_O_2_ concentration on the TOC removal rate has similar trends, regardless of the pH value. The TOC removal rate decreased slightly with the increase of H_2_O_2_. It can be concluded from the contour plots that the optimum region of the TOC removal rate is in the pH range of 2.48 to 3.05.

Figure 3c,d show the interaction effect of the Fe^2+^ and H_2_O_2_ concentration on the TOC removal rate. As can be seen in the plots, the increase of the Fe^2+^ concentration leads to an increase in the TOC removal rate. The ferrous ion acts as a catalytic agent in the decomposition of hydrogen peroxide. The increase in the initial concentration of the ferrous ion leads to more decomposition of the hydrogen peroxide and an increase in the degradation rate. We can see from the contour plots (Figure 3d) that the TOC removal rate is larger than 91.5% in the Fe^2+^ concentration range of 4.4–6.0 g∙L^−1^ either at a low or high level of H_2_O_2_ dosage. Therefore, it can be concluded that the increasing H_2_O_2_ concentration gradually decreases the TOC removal rate. Increasing the H_2_O_2_ concentration may promote an inhibitory effect by the hydroxyl radicals scavenging (Equation (4)) and the formation of another radical (HO_2_), which has an oxidation potential considerably smaller than HO**^.^** [21].

Figure 3e,f show the interaction effect of the pH and Fe^2+^ concentration on the TOC removal rate. For a pH value below 2.4, the amount of soluble iron Fe^3+^ decreases, inhibiting the radical OH formation [33]. pH values above 3.0 lead to the precipitation of iron hydroxides, inhibiting both the regeneration of the active species of Fe^2+^ and the formation of hydroxyl radicals. The contour plots show that the optimum region for the TOC removal rate is in the pH range of 2.6–2.7 and the Fe^2+^ concentration is in the range of 5.8–6.0 mg∙L^−1^, respectively.

### 3.5. The Prediction of the Optimum Condition of TOC Removal

To confirm the model’s adequacy for predicting the maximum removal of TOC (response function), we carried out a new experiment using the optimum levels, as shown in the Table 4. The result from Table 4 shows that there is a good agreement between the predictive and experimental results at the optimum levels, giving a high validity of the model.

## 4. Conclusions

The photo-Fenton process was found to be an efficient method for the treatment of aqueous solution contaminated by Tylosin antibiotic. The Response Surface Methodology (RSM) based on Central Composite Design (CCD) was used to evaluate and to optimize the effect of the hydrogen peroxide, initial pH and ferrous ion concentration. It was found that TOC removal increases with the increase of the ferrous ion concentration and decreases for pH values outside the range of 2.48 to 3.05. The TOC removal rate decreases slightly with the increase of the H_2_O_2_ concentration. The combination of RSM based on CCD proved to be a powerful tool in the optimization of the photo-Fenton reaction. The optimal conditions found for the Fenton reaction were, H_2_O_2_ 0.4 g∙L^−1^, pH range value 2.6, and Fe^2+^ 6 mg∙L^−1^; by using optimized values, the degradation reached 97.1%. Results were in good agreement with the ones predicted by the model.

## Figures and Tables

**Table 1:** Optimization of parameters, experimental range and level of independent variables, on photo-Fenton degradation of Tylosin.

Range and Level					
Independent variable	−α	−1	0	+1	+α
H_2_O_2_ (*X*_1_, mg∙L^−1^)	0.132	0.2	0.3	0.4	0.468
pH (*X*_2_)	1.89	2.3	2.9	3.5	3.9
Fe^2+^ Concentration (*X*_3_, mg∙L^−1^)	0.64	2	4	6	7.36

α = 1.68 (star or axial point for orthogonal CCD in the case of three independent variables) and their actual values were rounded.

**Table 2:** Experimental designs of the five levels and their experimental results and predictive values.

Run Number	*X*_1_ (H_2_O_2_)	*X*_2_ (pH)	*X*_3_ (Fe^2+^)	TOC Removal (%)	
				Observed	Predicted
1	0.2	2.3	2	74.83	73.13
2	0.4	2.3	2	65.01	68.36
3	0.2	3.5	2	55.68	56.51
4	0.2	2.3	6	85.92	83.45
5	0.4	3.5	2	31.15	34.85
6	0.4	2.3	6	96.04	96.44
7	0.2	3.5	6	71.24	69.12
8	0.4	3.5	6	62.29	65.22
9	0.13	2.9	4	87.28	91.12
10	0.46	2.9	4	89.42	83.83
11	0.3	1.92	4	61.03	61.87
12	0.3	3.88	4	24.22	21.64
13	0.3	2.9	0.73	65.29	62.21
14	0.3	2.9	7.26	95.1	96.44
15	0.3	2.9	4	88.15	88.27
16	0.3	2.9	4	88.63	88.27
17	0.3	2.9	4	88.93	88.27
18	0.3	2.9	4	88.45	88.27
19	0.3	2.9	4	87.85	88.27
20	0.3	2.9	4	88.32	88.36

**Table 3:** ANOVA for the response surface quadratic model.

Source	Sum of Squares	Degree of Freedom	Mean Squares	*F* Value	*p*-Value	Remark
Model	7698.9	9	855.4	75.7	<0.001	Significant
*A*_1_	66.1	1	66.1	5.6	0.038	Significant
*A*_2_	1957.4	1	1957.4	166.8	<0.001	Significant
*A*_3_	1417.9	1	1417.9	120.9	<0.001	Significant
*A*_12_	142.6	1	142.6	12.1	0.005	Significant
*A*_13_	157.7	1	157.7	13.4	0.005	Significant
*A*_23_	2.6	1	2.6	0.2	0.640	–
*A*_11_	0.0	1	0.0	0.0	0.7581	–
*A*_22_	3901.9	1	3901.9	332.6	<0.001	Significant
*A*_33_	126.9	1	126.9	10.8	0.005	Significant
Residual	112.9	10	11.3	–	–	–
Pure error	0.4	5	0.0913	–	–	–
Total	7811.8	19	411.147	–	–	–
–	*R*^2^ = 0.986	*R*^2^_adj_ = 0.973	–	–	–	–

*p* < 0.05 is considered as significant.

**Table 4:** Comparison of the predictive and the experimental result optimum values of TOC removal.

Parameter	Optimum Value	TOC Removal (%)	
		Predictive	Experimental
*X*_1_ (H_2_O_2_, g/L)	0.4	100	97.1
*X*_2_ (pH)	2.6	–	–
*X*_3_ (Fe^2+^, mg/L)	6	–	–

## Abbreviations

The following abbreviations are used in this:

RSM	Response Surface Methodology
CCD	Central Composite Design
TOC	Total Organic Carbon
ANOVA	Analysis Of Variance
pH	Hydrogen potential
UV	Ultraviolet
A_0_	Coefficient constant
A_ii_	Coefficient of the quadratic term
A_ij_	Interaction coefficient
TOC_0_	Total Organic Carbon in the beginning (mg/L)
TOC_F_	Total Organic Carbon in the end (mg/L)
Df	Degree of freedom
F	Fisher-Snedecor
R^2^	Determination coefficient (correlation coefficient)
R^2^_adj_	Adjusted coefficients of determination
